# Illusory Tactile Motion Perception: An Analog of the Visual Filehne Illusion

**DOI:** 10.1038/srep14584

**Published:** 2015-09-28

**Authors:** Alessandro Moscatelli, Vincent Hayward, Mark Wexler, Marc O. Ernst

**Affiliations:** 1 Department of Cognitive Neuroscience, University of Bielefeld, Bielefeld, Germany; 2 Cognitive Interaction Technology Centre of Excellence, University of Bielefeld, Bielefeld, Germany; 3 Sorbonne Universités, UPMC Univ Paris 06, UMR 7222, ISIR, F-75005, Paris, France; 4 CNRS, UMR 7222, ISIR, F-75005, Paris, France; 5 Laboratoire Psychologie de la Perception and CNRS, Université Paris Descartes, F-75006 Paris, France; 6 Multisensory Perception and Action Group, Max Planck Institute for Biological Cybernetics, Tübingen, Germany

## Abstract

We continually move our body and our eyes when exploring the world, causing our sensory surfaces, the skin and the retina, to move relative to external objects. In order to estimate object motion consistently, an ideal observer would transform estimates of motion acquired from the sensory surface into fixed, world-centered estimates, by taking the motion of the sensor into account. This ability is referred to as spatial constancy. Human vision does not follow this rule strictly and is therefore subject to perceptual illusions during eye movements, where immobile objects can appear to move. Here, we investigated whether one of these, the Filehne illusion, had a counterpart in touch. To this end, observers estimated the movement of a surface from tactile slip, with a moving or with a stationary finger. We found the perceived movement of the surface to be biased if the surface was sensed while moving. This effect exemplifies a failure of spatial constancy that is similar to the Filehne illusion in vision. We quantified this illusion by using a Bayesian model with a prior for stationarity, applied previously in vision. The analogy between vision and touch points to a modality-independent solution to the spatial constancy problem.

Spatial constancy refers to the ability of the perceptual system to gain stable estimates of the spatial configuration of external objects, even when the body—and thus the sensory receptors with which the objects are detected—is in motion^1^. To achieve spatial constancy, touch, like vision, requires movement-dependent transformations from somatotopic or retinotopic coordinates to world-centered coordinates. That is, an ideal observer should sum the relative motion measured on the sensory surface, the skin or the retina, with the movement of the sensor, in order to estimate the movement of the object in world-centered coordinates. The transformations actually performed by human vision differ from strict velocity summation, leading to biased estimates of object motion during eye movements. For instance, a stationary background appears to move in the direction opposite to that of the gaze during smooth ocular pursuit, a failure of spatial constancy known as the Filehne illusion^2^.

The perceptual system often integrates sensory measurements with prior knowledge about the world, so as to increase the precision of the combined estimate^3^,^4^. However, either an occasional mismatch between this prior knowledge and the actual status of physical objects or an unexpected change in sensory noise might lead to biases in the combined estimate and to perceptual illusions. Models invoking a prior for stationarity have been proposed to explain the Filehne illusion^5^,^6^, as well as analogous visual illusions for immobile^3^,^4^,^7^ and moving^8^,^9^ observers alike. According to these models, the observer assumes *a priori* that external objects are world-stationary. This assumption reflects our common experience that inanimate objects around us are usually at rest or in slow motion. A Bayesian model assuming a prior for stationarity predicts the Filehne illusion^5^, as follows. When pursuing a target stimulus, observers estimate the velocity of the background as the sum of the target velocity and the relative velocity between target and background. The target velocity is estimated from eye movement and the relative velocity is estimated from retinal slip. In both cases, the sensory measurements are combined with a static prior reflecting a prior belief that the world is stationary. Both sensory measurements are contaminated with different amount of noise and, hence, are also differentially affected by the prior. The differences in noise associated to retinal and extra-retinal inputs thus generate the illusion when the two are combined.

Does a similar motion illusion also occur in touch? It is a common belief that the sense of touch is less prone to illusions than vision. In common language, real objects are “tangible” while mirages are indeed visible but not touchable. In accordance with that, many philosophers have argued that the sense of touch provides us with a more direct window onto the world^10^,^11^, and consequently should be less prone to illusions. To test this centuries-old intuition that touch is better at providing spatial constancy than vision, we have investigated whether there is an analog of the Filehne illusion in touch. We determined that the perceived motion direction of a movable surface was biased during a pursuit hand movement, suggesting a failure of spatial constancy similar in touch as in vision. The analogy between the two sensory modalities points to a modality-independent solution to the spatial constancy problem.

## Results

**Figure 1:**
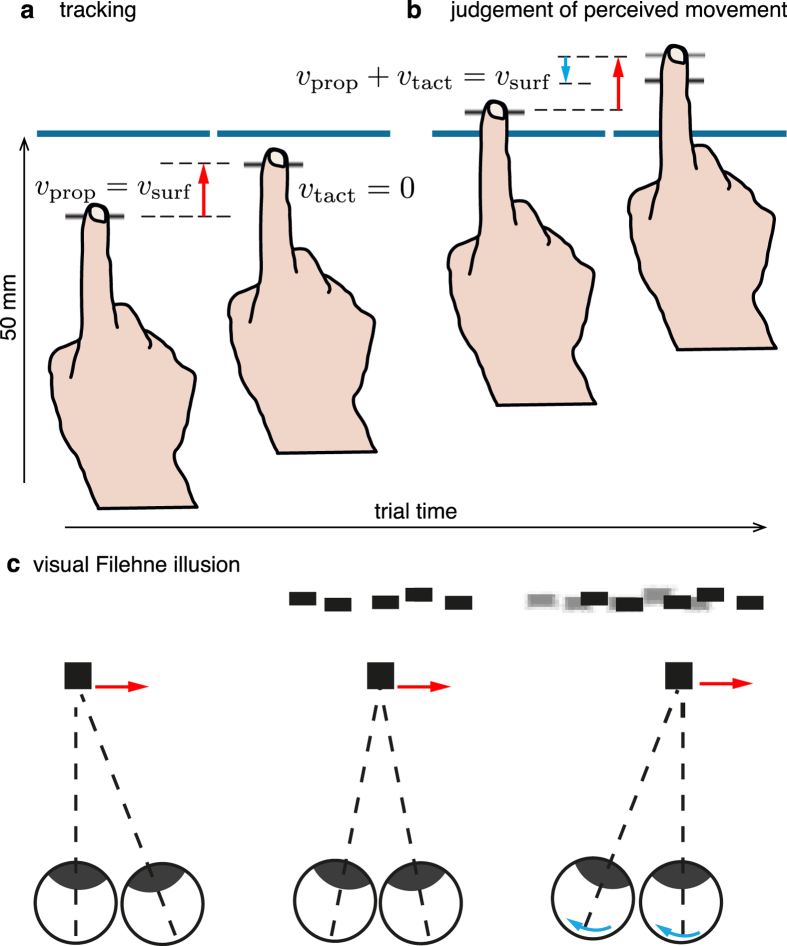
Procedure (main task). (**a**) Observers tracked a moving ridge in the proximal to distal direction. In the tracking phase of the stimulus, *v*_prop_ (the red arrow) provides an estimate of the initial surface’s velocity. (**b**) The velocity of the surface changed in the distal portion of the workspace, causing *v*_tact_ (the light blue arrow) to deviate from zero. The observer, moving his or her hand at the same speed, reported the perceived direction of motion with respect to the world reference frame. (**c**) For comparison, the Filehne illusion in vision. The red and light blue arrows represent the target speed (estimated from eye pursuit) and the relative speed between target and background (estimated from retinal slip), respectively. Figure in panel c is adapted with permission from Ernst (2010).^6^

Observers estimated the direction of motion of a movable surface, either during a manual pursuit movement (*main task*) or with the finger stationary (*control task*). The experimental procedures are represented in Fig. 1 and in the Supplemental Video. In the main task, observers tracked a ridged virtual surface that moved at a speed of 10 mm/s away from him or her in the horizontal plane (Fig. 1a). The surface was simulated by means of a tactile display (Latero, Tactile Labs, Inc.) that made it possible to modify its speed instantaneously. We simulated the movement of the ridged surface on the skin by oscillating in sequence the pins of the display (Methods). This generated a vivid sensation of tactile apparent motion for any speed of the virtual ridge. Following a displacement of 50 mm, the velocity of the surface changed suddenly but the observer was instructed to continue moving his or her finger at a constant speed, inducing a relative motion between the finger and the surface (Fig. 1b). At this point, observers judged whether the virtual surface moved toward or away from him/her. That is, they judged the velocity vector of the surface in an absolute reference frame. We coded the velocity of the surface *v*_surf_ > 0 if directed away from the participant, and *v*_surf_ < 0 otherwise. Maintaining a steady finger velocity past the transition was accomplished effortlessly and accurately (see Supplemental Data). This paradigm combined a tracking task with a perceptual task. The observers had to account for the velocity of their finger measured from proprioception, *v*_prop_, and for the relative velocity sensed by the cutaneous touch, *v*_tact_, in order to estimate the velocity of the surface in a world reference frame, *v*_surf_. For an ideal observer performing the task veridically,


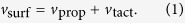


The control task was similar, with the only difference that the observers moved in the first part of the trial and then stopped and kept their finger stationary during the second part, *v*_prop_ = 0, *v*_tact_ = *v*_surf_, eliminating the necessity to account for the motion of the finger to estimate the world-centered surface motion.

**Figure 2:**
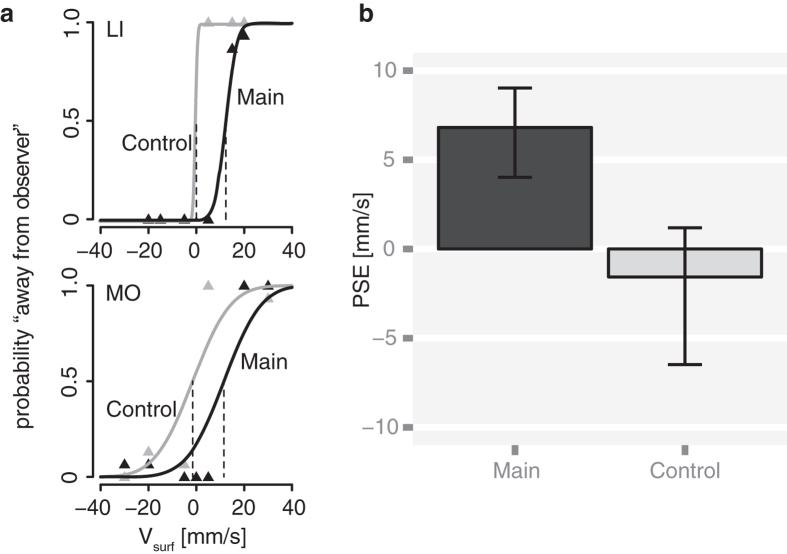
Experiment 1: Results. (**a**) Psychometric functions (*n* = 2: observers LI and MO). In both observers, the psychometric functions were centered around zero in the control task. Their PSE estimates were −1.5 ± 2.4 and −2.9 ± 1.0 mm/s (estimate ± SE). In the main task, the PSEs were significantly shifted toward positive values: the estimates were 11.5 ± 2.2 and 12 ± 1.1 mm/s. (**b**) PSE estimates (*n* = 10) were equal to 6.8 and −1.6 *mm*/*s* in the main and control task, respectively. Vertical bars represent the bootstrap-based 95% CIs.

Humans can estimate *v*_prop_^12^,^13^,^14^ and *v*_tact_^15^,^16^,^17^ accurately when presented in isolation. Therefore, observers could in principle estimate *v*_surf_ in the main task as well as in the control task, provided that they were able to correctly sum cutaneous and proprioceptive sources of information, as expressed by equation (1). Hence, the null hypothesis was that the estimate 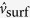 of the signal *v*_surf_ was accurate in the main as well as in the control task. An alternative hypothesis was that the integration of the two cues, by analogy with vision, produced a biased estimate in the main task but 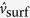 remained accurate in the control task. To test these hypotheses, we fitted the responses of each individual observer with psychometric functions of the form,


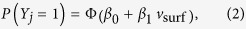


where Φ(⋅) is the cumulative normal distribution function. For each trial, *j*, the response variable *Y*_*j*_ had the value 1 if the observer reported that the surface was moving away from her, and 0 otherwise. We analyzed the data of all 10 observers using a Generalized Linear Mixed Model (GLMM), a hierarchical model extending the psychometric function to the group level^18^,^19^. Then, we evaluated the accuracy of the response to address our main question whether pursuit induced a bias in perceived motion in touch. To this end, we computed the *point of subjective equality* (PSE) and the 95% confidence intervals (CI) using the bootstrap method described in^19^. The PSE corresponds to the stimulus value yielding a response probability of 0.5 (dashed lines in Fig. 2a). Therefore, accurate responses should generate PSE estimates that do not differ significantly from zero.

Figure 2 shows the results of two representative observers (a) and the PSE estimates in the group data of all 10 observers (b). The PSE was significantly larger than zero in the main task, 6.8 ± 1.2 mm/s (estimate ±SE), while it did not significantly differ from zero in the control task, −1.6 ± 1.8 mm/s. The bootstrap-based 95% CIs ranged from −5.8 to 1.2 mm/s in the control task and from 4.3 to 9.1 mm/s in the main task (Fig. 2b). We computed the 95% CI of the difference in PSE between conditions to test if the two were statistically significant. This CI ranged from 5.7 to 12.1 mm/s. Crucially, the interval did not comprise zero, which means that the difference was statistically significant. The fact that the PSE in the main condition is significantly larger than zero and greater than in the control condition demonstrates the existence of a tactile Filehne illusion. The GLMM fit to the individual data is illustrated in the Supplemental Figure S2.

In summary, a stationary surface was perceived to be moving in space in the direction opposite to that of the movement of the finger. The tactile illusion described in this study is a putative equivalent of the visual Filehne illusion. In touch, as in vision, the perceived movement of a stimulus is biased during smooth pursuit of a target and a stationary background is perceived to be moving in the direction opposite to the movement of the target. The strong bias during active motion suggests, like in vision, a weak spatial constancy in touch. In vision, retinal motion during fixation needs to be slower by around 50% to achieve the perceived-speed match with pursued motion^5^. We estimated the gain factor to quantify the illusion in touch, as follows. If PSE_main_ is the PSE of the main task (estimated as 6.8 mm/s) and 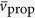 is the average finger velocity (calculated to be 10.8 mm/s), then the ‘haptic Filehne gain’ is 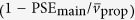. This quantity was estimated to be 1 − (6.8/10.8) ≈ 0.4. Therefore for a biased observer, equation (1) becomes,


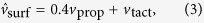


where 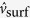 stands for the perceived surface velocity. In accordance with equation (3), the PSE in the main condition was smaller than the mean finger speed and the different was statistically significant, which implies that finger motion was taken into account in judging surface velocity, but with a gain smaller than one. Our finding implies that, on the average, observers perceived a stationary surface as moving opposite to their finger movement, with a speed equal to roughly 60% of the finger movement, which is comparable to the size of the illusion in vision, but slightly stronger.

### Model

In vision, a Bayesian model assuming the existence of a prior for stationarity can predict the occurrence of the Filehne illusion^5^,^6^. When pursuing a target stimulus, observers estimate the velocity of the background as the sum of the target velocity and the relative velocity between the target and the background (Fig. 1c). The target velocity is estimated from eye movement (red arrow) and the relative velocity is estimated from retinal slip (light blue arrow). In both cases, the sensory measurements are combined with a static prior, *S*, reflecting a prior belief that, due to ubiquitous friction force, object around us are more likely at rest rather than in motion^3^,^4^,^5^,^6^,^8^,^9^. The sensory measurements are associated to different noise levels, hence are differentially affected by the prior *S*. The different noise associated to retinal and extra-retinal inputs generate the illusion when they are combined.

**Figure 3:**
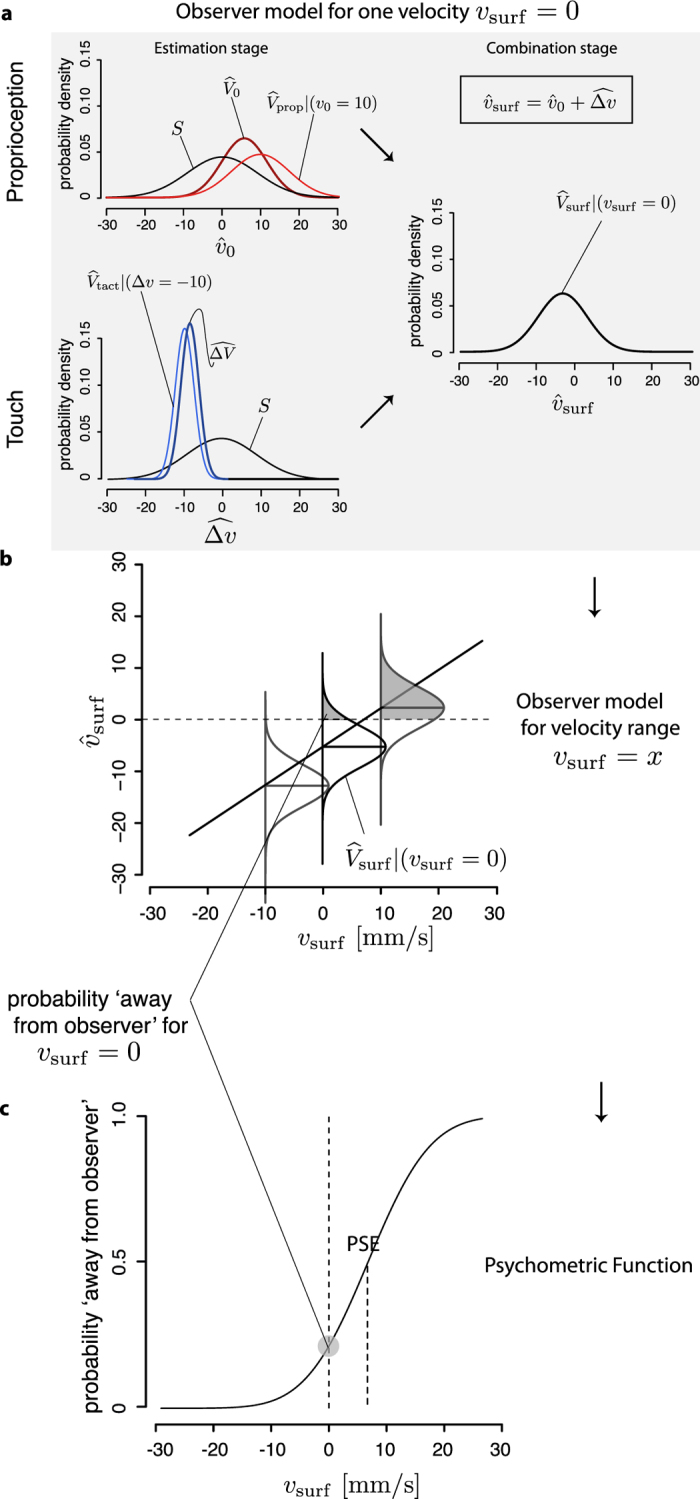
Bayesian Model. 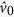
 is the estimate of the initial velocity (from hand pursuit), 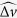 is the estimate of the velocity change (from tactile slip), 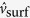 is the world-centered estimated of the surface velocity, *S* is the static prior. (**a**) The model transforms the difference in noise between proprioception and touch in a difference in accuracy (Estimation stage). The combination of the two estimates (Combination stage) generates a bias. (**b**) We extended the model to a continuous interval of the physical velocity, by modeling the fused estimate, 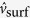, as a linear function of the physical velocity, *v*_surf_, plus Gaussian noise. The parameters of the linear equation and the Gaussian noise are fully specified by the variance of the prior and of the two sensory measurements (Supplemental Data). (**c**) Finally, we related the observed model to the measured psychometric function defined in equation (2) by posing *Y*_*j*_ = 1 if 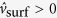 and *Y*_*j*_ = 0 otherwise.

A crude analogy between vision and touch might consider that the finger is like the eye and the fingertip skin is like the retina. It can be independently established that the proprioceptive inputs from the upper limb are noisier than the tactile inputs, the difference in noise reported in the literature ranges from 10% to 50%^12^,^13^,^14^,^17^. Thus, we wondered if the noise difference in the two sources of information could account for the observed effect. To this end, we fit our data to the model proposed in vision (Fig. 3). In the following discussion, a lower case letter denotes a random variable (e.g., 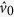 in Fig. 3a) and a capital letter a probability distribution (e.g., 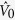).

Some adjustment were necessary to account for the differences between visual and tactile experimental paradigms. Freeman *et al.*^5^ assumed simultaneous processing of absolute and the relative velocities. Differently from vision, our stimuli had two distinct phases, the initial phase, where the finger pursued the surface and the movement on the skin was zero, and the subsequent phase where the movement could be non-zero. Therefore, we expressed our model as a sum of two terms, an estimate of the initial velocity of the surface and an estimate of its velocity change—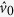 and 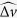, respectively. In order to estimate the initial and relative velocity, the observer fused the proper sensory measurement with the prior belief about the object’s motion (the Estimation stage in Fig. 3a). Since pursuit was accurate, the proprioceptive-based sensory measurement 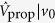 (Fig. 3a) is a proper substitute for *v*_0_. The notation ‘|*v*_0_’ stresses that the distribution is conditional to a specific value of the physical stimulus. Likewise, the tactile-based sensory measurement 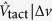 can be assumed to be an accurate substitute for Δ*v*, given that the velocity of the finger remained unchanged between the initial and the final portion of a trial (Supplemental Data). We assumed that each of the two sensory measurement was corrupted by zero-mean, normally-distributed noise. As in the study in vision^5^, we used a zero-centered, normal distribution to model the prior belief that the surface was stationary, the static prior *S*. Each of the two measurements was combined with the static prior to produce the estimate of the initial velocity and of the velocity change (the posterior distributions):


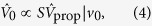



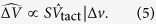


These are represented in Fig. 3a as the dark red and blue distributions. According to standard results in mathematical statistics, with a greater variance of the sensory inputs, comes a greater influence of the prior on the posterior estimates^20^,^21^. Since the proprioceptive inputs are noisier than the tactile inputs^12^,^13^,^14^,^17^, the stationarity prior has a different influence on each of the two posterior distributions. Therefore, the mean of 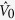 should be closer to zero than that of 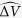.

Finally, we modeled the fused estimate of the velocity of the surface, 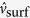, as the sum of its perceived initial velocity 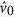 and of the perceived velocity increment 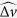 (the Combination stage in Fig. 3a),


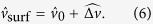


We assumed that the fused estimate 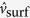 is the sum of two random samples from the posterior distributions 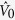 and 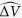. Due to the difference in mean between the posteriors, the estimated surface velocity, 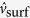, is attracted by *v*_tact_, producing the perceptual bias.

The model described above is conditional to a specific value of the physical stimuli, *v*_surf_ = 0 in Fig. 3a. It can be extended to a broader range of velocities in order to relate the model to empirical psychometric functions. To this end, we made the following assumptions: (i) In the sampled range of velocities, each of the two sensory measurements is an unbiased estimator of the corresponding physical stimulus. Therefore, the mean of each of the two likelihoods is a linear function of the physical stimulus, and corresponds to the identity line. According to^15^, for stimuli ranging from ten to hundred millimeters per seconds, a linear model described tactile velocity perception in humans reasonably well. (ii) The variance of the two sensory measurements varies slowly with speed, and can be approximated to a constant in the sampled range. (iii) The variance and the mean of the prior are constant. It follows from (i-iii) that 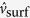 is a linear function of the physical velocity *v*_surf_ plus zero-mean Gaussian noise (Fig. 3b). Note that, for each value *x* of *v*_surf_, the perceived velocity has the conditional distribution 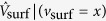. Finally, we related the observed model to the measured psychometric function defined in equation (2) by posing *Y*_*j*_ = 1 if 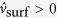 and *Y*_*j*_ = 0 otherwise (Fig. 3c). Therefore, the probability of responding “away”, *P*(*Y*_*j*_ = 1), is equal to the probability that the estimated surface is larger than zero, 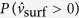.

We fit the Bayesian model via maximum likelihood and used a parametric bootstrap procedure to estimate its parameters and the PSE (Experimental Procedures). In the main task, the Bayesian model yielded an estimated PSE equal to 7.2 mm/s (95% CI 1.8–10.4 mm/s), which is close to the estimate provided by the GLMM (6.8 mm/s). Furthermore, the model predicted an unbiased response (PSE = 0 mm/s) in the control task, where the hand was stationary. Accordingly, the PSE estimated from the GLMM was not significantly different from zero (95% CIs ranging from −5.8 to 1.2 mm/s). In summary, the predictions of the Bayesian model were comparable with those of the descriptive model.

The Bayesian hypothesis assumed that the tactile Filehne gain was caused by a difference in noise between proprioception and touch. To test if this was the case, we predicted separately for proprioception and touch the noise of the response, and compared these predictions to the values reported in the literature. We adopted the same Bayesian model to predict the response of the observer in two simulated unimodal discrimination tasks. We assumed that the signal from proprioception was discarded during the ideal tactile discrimination task, and *vice versa*. Therefore, the slope of each psychometric function depended on the variance of the prior and on the variance of the respective sensory measurement, either from proprioception or from touch. The values of the slopes predicted by the model were equal to 0.02 for proprioception and 0.06 for touch. This corresponds to a *Just Noticeable Difference* (JND) equal to 33.75 mm/s and 11.25 mm/s, respectively. Thus, in order to generate the observed bias, proprioceptive velocity discrimination had to be three-fold noisier than tactile velocity discrimination. However, comparing the results across different studies^12^,^13^,^14^,^16^,^17^, the response noise in cutaneous touch was no more than 50% larger than in proprioception.

The relationship between bias and noise predicted by the model only holds if we assume that the prior for the absolute motion is the same as the prior for the motion change. If we introduce in the Estimation stage (Fig. 3a) two priors, *S*_prop_ and *S*_tact_, with zero mean but different variances, the model would fit the data, i.e. it would fit the observed bias even when the tactile and the proprioceptive posterior noise are comparable. Relaxing the assumption that the two variances are the same is reasonable as the prior statistics of the initial velocity and velocity change will likely differ in the spread of the distribution, although they will be equally distributed for left and right motion and thus centered at zero. Still, the model predicts a reduction of the motion bias if tactile variance increases, with other parameters being unchanged, because in such a situation the prior will have more influence on the tactile estimate. Hence, we ran a second experiment to verify this prediction.

### Testing Model Predictions: Tactile Noise Modulates the Illusion

In the first experiment, we elicited a tactile motion illusion akin to the Filehne illusion in vision. A Bayesian model implying a stationarity prior successfully predicted the illusion in vision. It would be possible to extend the model to the tactile task by assuming two zero-mean priors, each with different variance, for the estimate of the initial velocity and the velocity change. According to the model, the illusion should be smaller as the tactile measurement becomes noisier, with other parameters being unchanged. We tested this prediction in the following experiment.

A modulation of the signal contrast is a well-established procedure to modulate perceptual noise in vision^4^. Here we changed the amplitude of oscillation of the pins in order to modulate the tactile signal-to-noise ratio, i.e. the tactile contrast. The experimental procedure was the same as in the main task of the first experiment (Fig. 1a,b). Observers (N = 10) pursued the moving surface in the first half of the trial and then kept moving in the second half. After each trial, they reported whether the surface moved towards or away from them in the second half of the trial. This time, we modulated the reliability of the tactile stimulus in the second half of the trial by varying the oscillation amplitude of the pins. The amplitude was always equal to 0.1 mm in the first half of the trial during pursuing, whereas it was either 0.1 mm or 0.04 mm in the second half (high and low amplitude condition, respectively).

**Figure 4:**
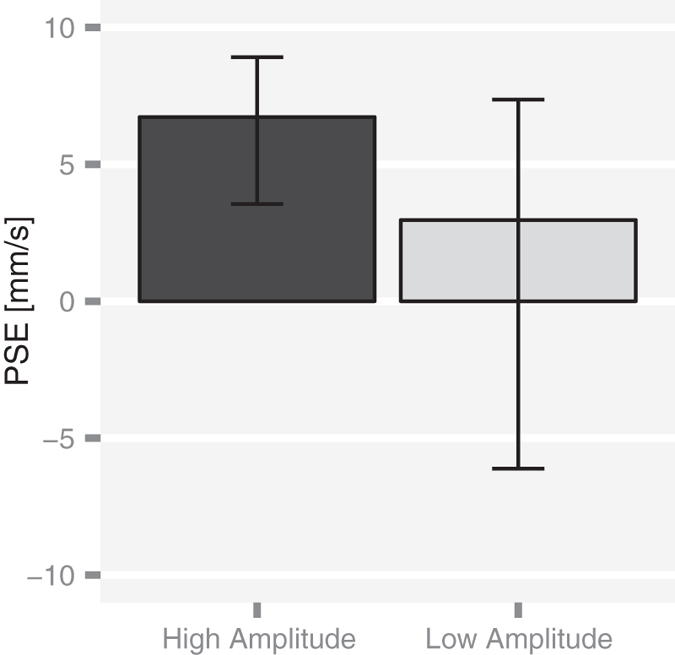
Experiment 2: Results. PSE estimates (*n* = 10) were equal to 6.7 mm/s (95% CI from 3.9 to 8.8 mm/s) for the high oscillation amplitude and 3.0 mm/s (95% CI from −6.6 to 7.4 mm/s) for the low oscillation amplitude. Vertical bars represent the bootstrap-based 95% CIs.

We fit the data of Experiment 2 with the GLMM model (8). First, we estimated the perceptual noise from the fixed slope of the model (see Methods). As expected, the response was significantly noisier in the low amplitude compared to the high amplitude condition (*p* < 0.001). The Bayesian model predicted that the increase in the tactile noise should lead to a smaller motion bias. That is, the PSE should be closer to zero. Results were consistent with this prediction (Fig. 4). The bias was about the same as in Experiment 1 for the high oscillation amplitude (PSE_high_ = 6.7; 95% CI from 3.9 to 8.8 mm/s) and non significantly different from zero for the low oscillation amplitude (PSE_low_ = 3.0; 95% CI from −6.6 to 7.4 mm/s). We used a a bootstrap procedure to test whether this difference in PSE between conditions, PSE_high_ − PSE_low_, was statistically significant. The estimated difference was equal to 3.76 mm/s, 95% CI ranging from 0.43 to 11.05 mm/s. Since its 95% CI did not include the zero, we concluded that the difference in PSE was statistically significant in accordance with the model prediction.

Finally we run a tactile velocity discrimination task to confirm that, in analogy to the modulation of the stimulus contrast in vision, modifying the oscillation amplitude of the pins modulated the tactile contract and thus the reliability of the tactile estimate (Experiment 3). In each trial, observers (N = 8) maintained the hand world-stationary and reported in which of two subsequent intervals the surface was moving faster. The amplitude of pin oscillation changed between trials and was either 0.1 mm or 0.04 mm. As in Experiment 2, the response was significantly noisier for the lower oscillation amplitude (*p* < 0.01). The estimated JND was 5.9 ± 0.7 mm/s for the high amplitude (JND ± Std. Error) and 8.3 ± 1.4 mm/s for the low amplitude. Refer to the Supplemental Data for further details on Experiments 2–3.

## Discussion

In the current study, we measured the perceived direction of a moving surface sensed with a hand that was either stationary or in motion. We observed a large bias when the hand was in motion, such that for the particular stimulus used a world-stationary surface would seem to move in the opposite direction as the hand, with a speed roughly equal to 60% of the speed of the hand. The reported illusion is surprising since we do not feel objects move in the direction opposite to the movement of a finger sliding on them. This discrepancy might be apparent only. The confidence that we have *a priori* that inanimate objects are world-stationary is strong since it is seldom the case that objects move when interacting with them with a light touch. Moreover, in natural conditions, vision often contributes powerfully to a fused estimate of movement during object exploration.

The phenomenon described here is putatively equivalent to the Filehne illusion in vision. Following^5^, we fit the data with a Bayesian model where the posterior estimates of the velocity of the external surface were obtained from the combination of the noisy measurements from proprioception and touch with a stationarity prior. The stationarity prior reflects our common experience that inanimate objects around us are usually at rest or in slow motion. Prior assumption that objects are usually world-stationary does not only affect motion perception, but it also has a strong influence on human reasoning and cognition. For millennia, from Ancient Greece to Middle Ages, human’s prescientific theories of motion were based on the idea that stationarity is the natural state of inanimate objects and motion is a temporary deviation from this natural state^22^. Medieval theorists believed that a projectile was moved by an internal force called *impetus* and as soon as this internal force dissipated the projectile would return to its natural, resting state. Nowadays, the idea that stationarity is the natural state of inanimate objects often remains as implicit thought in naïve physical reasoning^22^,^23^.

According to a Bayesian model, sensory noise plays a crucial role in generating the illusion. Sensory noise may arise both internally (e.g., due to the stochasticity of synaptic releases or to the chaotic dynamics of neural networks) and externally (e.g., due to the stimulus noise). An approximation in the computations performed by the nervous system, such as suboptimal inference, can also increase the sensory noise and contribute to the variability in the perceptual judgment^24^. In a Bayesian framework, the noisy sensory measurements and the prior knowledge are modeled as probability distributions^4^,^5^,^20^. This raises the question whether probability distributions can be represented by the activity of a population of neurons. Several studies^24^,^25^ proposed a probabilistic population code, where latent stochastic processes underlie the spike trains of each neuron and thus generate probability distributions. In a previous study^25^ the response distribution simulated from a probabilistic neural network was described reasonably well by a Poisson or by a Gaussian distribution, which justifies the Gaussian approximations in equation (5)–(4). Changes in the external noise modulate the variance of the response of the neural population^24^. Accordingly, in Experiment 2 we reduced the reliability of the tactile signal in order to increase the variance of the likelihood, which thereby increased the behavioral noise as was measured with the psychometric function.

The Bayesian model made three simplifying assumptions, namely (i) that the relationship between the perceived and the physical speed was locally linear, (ii) that the same prior accounted for both the relative and the absolute speed, and (iii) that the likelihoods were unbiased. Within the tested range of stimuli, the linearity assumption is in accordance with previous results in the literature of tactile speed perception^15^,^16^. At its core, our model assumed that the *probit* function of the response probability (i.e., the inverse function of a cumulative Gaussian function) was a linear function of the physical speed of the surface (S8). Essick *et al.*^16^ run a velocity discrimination task to test this assumption. Inspection of Fig. 4 of the original article reveals an excellent fit of the linear model to the data. Accordingly, within a velocity range of 10–100 mm/s, the perceived speed was approximated reasonably well by a linear function of the stimulus^15^. The linear assumption might fail when exposing the observer to a wider range of stimuli. For instance, for a stimulus ranging from 50 to 2560 mm/s a power function provided a better fit to the data^16^. However, we would like to stress that the assumption in our model is to provide a local approximation of the function within the limited range of velocities tested in the study. GLMMs (7)–(8), which rely on the same linearity assumption, provided a good fit to our data, as attested by the model plots of the individual participants in Supplemental Data.

The second assumption stated that the prior for the absolute velocity of the target (the initial velocity in our task) was the same as the prior for the velocity relative to the background (the velocity change). Assuming a single prior, the Bayesian model would predict a large difference in the sensory noise between proprioception and cutaneous touch. Several earlier studies have shown that this is not necessarily the case^12^,^13^,^14^,^17^. Introducing two zero-mean priors with a different variances in each of the two estimates would extend the model to fit datasets like ours, where the difference in JND between the two discrimination tasks is small. Such an extension would be reasonable since the statistics of the relative and absolute velocities, although centered on zero, as all directions are equally probable, will likely differ in their magnitude and thus variance. Still, the model predicts a reduction of the motion bias if tactile variance increases, with other parameters being unchanged. Accordingly, we found that increasing the external noise in touch reduced the strength of the illusion (Experiment 2).

Finally, the third assumption stated that the two likelihoods were unbiased and, therefore, the illusion arises uniquely from their difference in noise. However, results from the literature^26^,^27^,^28^ suggest that other factors, beyond the perceptual noise and the stationarity prior, might play a role in the illusion. For instance, it stands to reason that the nature of the surface on which the finger slips, real or virtual, can affect the estimated slip velocity^15^. Similarly, in vision the estimated speed of a moving background is affected by its spatial frequency, so that the higher is the frequency, the faster the estimated motion^27^. Since the noise of the velocity estimate in touch is also affected by the nature of the surface, it remains to be seen whether the biases observed with different surface textures can be explained with a Bayesian Model assuming unbiased estimates. Therefore, in our model we maintained the assumption of unbiased prior. Allowing for a biased likelihood for fitting our data would introduce two further parameters in the model (the mean of the two likelihoods), at the cost of a possible over-fitting. However, in future studies, the effect of the background texture and surface properties may need to be studied systematically in order to investigate whether tactile velocity estimation can be fully explained by this simple model assuming a stationarity prior or whether it has to be extended to allow for additional biases in the sensory estimates.

In conclusion, touch—like vision—shows rather poor spatial constancy^29^,^30^, leading to motion illusions during active hand movement, akin to the Filehne illusion. Motion processing shows other remarkable analogies between vision and touch. For example, in both sensory modalities, the perceived speed is affected by the spatial frequency of the stimulus^15^,^27^ and modulated by a motion after-effect^31^,^32^. The analogy between vision and touch is surprising, given the profound differences in the physics of the two signals and in the physiology of the two sensory modalities. Our study support the hypothesis that, despite the huge differences in stimulus encoding, vision and touch would share common mechanism of motion processing at a higher level of representation.

## Methods

### Participants

Twelve naïve participants took part in Experiment 1 (6/12 females, 26 ± 7 years old, mean ± SD). One observer produced a paradoxical response pattern in one condition, possibly due to a misunderstanding of the response coding. A second observer produced a constant response probability, which was at chance-level irrespectively of the stimulus. These two observers were excluded from further analysis. Ten naïve participants took part in Experiment 2 (5/10 females, 24 ± 4 years old). Eight naïve participants took part in Experiment 3 (4/8 females, 25 ± 5 years old).

The testing procedures were approved by the “Comité de protection des personnes Ile-de-France II” permit 2011-06-16 (IRB registration 1072), in accordance with the guidelines of the Declaration of Helsinki for research involving human subjects. Informed written consent was obtained from all participants involved in the study.

### Stimuli and Procedure

The stimuli were produced with the Latero stimulator^33^ (Tactile Labs, tactilelabs.com). The active surface consisted of an 8 by 8 array of laterally moving pins, actuated by miniature piezoelectric bending motors. The active area of the display was 1.2 cm^2^. We simulated the movement of a ridged surface on the skin by actuating the rows in sequence with a 20 Hz oscillatory signal generating a vivid sensation of tactile apparent motion for any speed of the virtual ridge. The amplitude of the oscillation was 0.1 mm. The position, 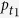 at time *t* of the actuated row was related to the position of the previously actuated row by,


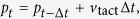


where *v*_tact_ was the desired apparent velocity of the surface with respect to the skin, and Δ*t* was the refresh period (1.5 ms) of the display. The distance between the ridges of the simulated surface was equal to 12.8 mm, corresponding to the distance between the first and the last row of the array plus 1.6 mm. This ensured that only a single moving ridge was felt at any given time. The vibration of the next row started immediately when stopping the prior row without cross fading. The display was supported by a carriage sliding on a smooth Teflon surface. The position of the carriage was measured with an accuracy of 50 m from which *v*_prop_ could be precisely determined.

Experiment 1 consisted of a main and a control task. Each of the two tasks was tested in a separate block within the session and the order of the two tasks was counterbalanced between observers. Training sessions preceded the experimental sessions. Observers sat in a dimly lit room with the right arm parallel to the motion path. Pink auditory noise was delivered to the observers via earphones throughout each experimental session in order to mask external sounds. In the main task (Fig. 1), observers touched the display with the tip of the right index finger and felt the ridged stimulus moving in the distal direction. They were instructed to move their hand in order to track it. The speed of the ridge had a fixed value of 10 mm/s during the first 50 mm of finger displacement. Past this distance the velocity changed suddenly in a pseudo random fashion (velocities ranging from −30 to 30 mm/s, including 0). A trial ended when the finger reached a total displacement of 100 mm. After each trial, the observer reported whether the virtual surface was moving away or towards her. This corresponds to the direction of the red arrow in Fig. 1b. The control task was similar to the main task, with the difference that the observer brought the hand to rest in the second portion of the path. The observer first performed a tracking movement as in the main task but stopped moving past a visible marker, so that *v*_surf_ = *v*_tact_. The trial terminated seven seconds after having crossed the border so that the presentation time was comparable to the presentation time in the main experimental task. The velocity range was also from −30 to 30 mm/s, not including zero. Each experimental session consisted of 195 trials (105 in the main and 90 in the control task).

The experimental procedure was the nearly same in Experiment 2. This time, the amplitude of the oscillation of the pins was equal to 0.1 mm in the first half of the trial, while pursuing, whereas it was either 0.1 mm (high amplitude) or 0.04 mm (low amplitude) in the second half. The experimental session consisted of a single block of 200 trials. High and low oscillation amplitude were randomly intermixed within the same session.

### Descriptive model

For each observer, we recorded the proportion of “away-from-me” responses over the total number of responses, as a function of the actual value of *v*_surf_. We modeled participant’s responses using model (2). Next, we performed a group analysis by means of a generalized linear mixed model (GLMM, see^18^,^19^). The GLMM is an extension of the general linear model (i.e., the psychometric function) to clustered data, here the repeated responses of each single observer. The GLMM is a hierarchical model, including fixed and random-effect parameters. For a single experimental condition (either main or control task) the model was,


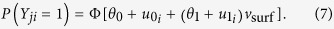


Similarly as in model (2), the response variable on the left side of the equation is the the probability of responding “away-from-me”. The fixed-effect parameters *θ*_0_ and *θ*_1_ are the fixed intercept and the fixed slope, accounting for the effect of the experimental variable *v*_surf_. The random-effect parameters 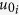 and 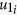 accounted for the variability among different observers. Having two random-effect parameters, the model assumed that in each observer, *i*, the intercept and the slope of the response function are sampled from a bivariate Gaussian distribution. We fit simultaneously the main and the control condition using a multivariable GLMM^18^,^19^. The routine lme4 of the R programming language was employed to fit the model^34^. From model (7) we estimated the PSE and the 95% confidence interval in the two experimental conditions, as described in^19^. The distribution of the difference in PSE was estimated with the same bootstrap procedure.

We fit data of Experiment 2 with the a GLMM. For each oscillation amplitude, the model had the form:


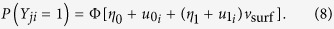


We fit simultaneously the high and low amplitude condition using a multivariable GLMM (for simplicity we reported in equation (8) the univariable version of the model, referred to a single oscillation amplitude). The fixed slope parameters *η*_1_ estimates the perceptual noise, the higher the slope the smaller the noise. We tested whether the parameter was significantly different between the high and low amplitude using the Likelihood Ratio Test. As for Experiment 1, we estimated the PSE and the 95% confidence interval in the two amplitude condition, as well as the distribution of the PSE difference.

### Bayesian model

The Bayesian model as formulated in S8 (Supplemental Data) belongs to the family of general nonlinear model. The right-hand-side of the equation is a nonlinear function of the two predictors *v*_prop_ and *v*_tact_ and it has three free parameters corresponding to the variance of the prior, and the variance of the two likelihoods. We used the R function gnm^35^ to fit S8 (Supplemental Data) to the data. Maximum likelihood estimation was employed to fit the model to the data produced by each participant and a parametric bootstrap procedure was used to estimate the parameters for the whole population as follows: In the main and the control task, the responses were simulated from a binomial distribution, (*n*, *p*), where *p* was Φ(*b*_0_ + *b*_1_*v*_surf_) and *n* was equal to the number of trial repetitions. The parameter *b*_1_ was equal to the slope either in the control or in the main task, as estimated in the descriptive model. We set the intercept *b*_0_ assuming that the responses were unbiased in the control task (−*b*_0_/*b*_1_ = 0 mm/s) and positively biased in the moving task (−*b*_0_/*b*_1_ = 6.8 mm/s). We repeated the simulation to provide one thousand bootstrap repetitions and fit the model equation S8 (Supplemental Data) to each simulated dataset. When the difference between the likelihood and the prior variances is large, a large change in the parameters produces only a small change in the corresponding weighting factor jeopardizing convergence. The parameters of the prior were therefore constrained during the fitting procedure. The estimated likelihood variances were 34 mm^2^/s^2^ for the proprioceptive measurement (95% CI 12–225 mm^2^/s^2^) and 11 mm^2^/s^2^ for the cutaneous measurement (95% CI 8–14 mm^2^/s^2^). We verified that constraining the variance of prior to different values did not modify the estimated ratio between kinesthetic and tactile noise. The GLMM analysis revealed a significant difference in slope between the control and the main task, the slope being significantly larger in the former (*p* < 0.001). To account for that, we assumed that the observer discarded proprioceptive information when the finger was stationary (i.e. in the control task). Freeman *et al.*^5^ assumed that the variance of the two sensory measurement was a non-linear function of the velocity. This would also account for the difference in slope between our two tasks, however would require adding four more parameters to the model.

## Additional Information

**How to cite this article**: Moscatelli, A. *et al.* Illusory Tactile Motion Perception: An Analog of the Visual Filehne Illusion. *Sci. Rep.* **5**, 14584; doi: 10.1038/srep14584 (2015).

## Supplementary Material

Supplemental Video

Supplementary Information
