# Supplementary material for: Illusory Tactile Motion Perception: An Analog of the Visual Filehne Illusion
Source: Sci Rep. 2015 Sep 28;5:14584. doi: 10.1038/srep14584 (PMC4585937; doi:10.1038/srep14584)
Supplement: Supplementary Information [file srep14584-s2.pdf]

Illusory Tactile Motion Perception: An Analog of the Visual Filehne Illusion (Supplementary Information)

Alessandro Moscatelli, Vincent Hayward, Mark Wexler, Marc O. Ernst

Experiment 1: Analysis of the Motor Behavior

The task required participants to track a moving surface in the first half of the trial. In the second half of the presentation the velocity of the tracked surface changes, while participants were asked to keep the velocity of the hand roughly constant. The analysis of the motor response aimed at evaluating if participant attained to these task requirements. In both the main and the control task, the value of v_{surf} during the tactile pursuit (ie., the first half of the path) was equal to 10 mm/s. Analyzing the motor response, we found that In this first half, the grand mean of v_{prop} was 11.2 mm/s in the main task and 11.4 mm/s in the control task. In the main task, in the second half of the trials, the grand mean of v_{prop} was 10.8 mm/s. Therefore, in different motion condition, the discrepancy between the tracked v_{surf} and v_{prop} was relatively small, with a slightly tendency to move faster then the tracking surface. To confirm this, we applied a linear mixed model to parametrically test whether the participants in the main task were able to keep the velocity of motion constant in the two halves of the trials [8],

$$v_{\text{prop}_2} = u_0 + v_{\text{prop}_1}(\gamma + u_1). \tag{S1}$$

This model assumes that the finger velocity in the second part of the trial (unconstrained movement, v_{prop_2}) is a linear function of the velocity in the first half of the of the trial (guided movement, v_{prop_1}). The fitted model should be close to the identity line, if participants were able to keep the hand velocity constant in the two parts of the trial. The difference between participants was modeled as random noise (the random intercept u_0 and the random slope u_1). Notice that there is only one fixed-effect parameter γ to account for the effect of the guided movement. That is, we constrained the fixed intercept to zero. Supplementary Fig. S1 shows the model fit for the ten participants tested. For all of them, there was a high correlation between the two velocities in the different parts of the trial. Accordingly, the estimate of the slope parameter γ was 0.86 ± 0.05 ($\gamma \pm$SEM). The parameter is highly significantly different from zero ($p < 0.001$). As shown in Supplementary Fig. S1, in six participants out of ten, both the regression line and the data cloud were close to the ideal (i.e, the data cloud clustered around the point 10,10 in the plot). Looking at the relative perceptual estimates in the discrimination task, in all these participants there was a significant difference between the control and

the main task, and in five of them there was an large bias in in the main task, which cannot be simply explained as an imprecision in the motor behavior.

In conclusion, the analysis of the motor behavior showed that participants were able to move the hand at the velocity required by the task. Therefore, the perceptual bias reported in the article is unlikely to originated from systematic errors in the motor behavior.

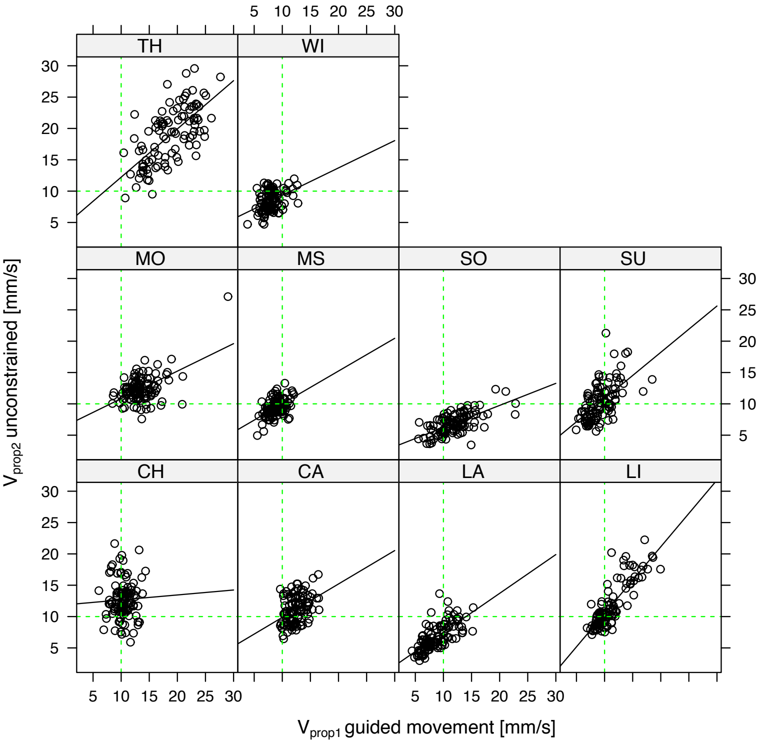

Supplementary Figure S1: The motor behavior and the predictions of the linear mixed model described in Equation (S1). Each panel shows the raw data and the fitted model line. The cross between the two green dashed lines it the ideal response, where the velocity in both, the pursuit of the first half and the unconstrained movement of the second half is equal to 10 mm/s

Supplemental Video

The Supplemental Video illustrates the experimental procedure. In the main task, observers tracked a ridged virtual surface that moved at a speed of 10 mm/s away from him or her in the horizontal plane. The surface was simulated by means of a tactile display (Latero, Tactile Labs, Inc.). We simulated the movement of the ridged surface on the skin by oscillating in sequence the pins of the display. This generated a vivid sensation of tactile apparent motion for any speed of the virtual ridge. Following a displacement

of 50 mm, the velocity of the surface changed suddenly but the observer was instructed to continue moving his or her finger at a constant speed, inducing a relative motion between the finger and the surface. At this point, observers judged whether the virtual surface moved toward or away from him/her. The control task was similar, with the only difference that the observers moved in the first part of the trial and then stopped and kept their finger stationary during the second part, eliminating the necessity to account for the motion of the finger to estimate the world-centered surface motion.

Experiments 1-3: Supplemental Figures and Data

Generalized Linear Mixed Models provides both predictions on the experimental effects, which are assumed to be systematic across participants, and an estimate of the variability between participants. Participant-specific adjustments (referred as conditional modes, as they are conditional to the fixed and random effect parameter fit) are also provided. By combining fixed parameters and conditional modes, it is possible to get and visualize GLMM predictions at the single-participant level. These are extremely useful to evaluate the model fit to the data. GLMM fit to experiment 1-3 are provided below. Figure caption provides further details on the analysis (experiment 1) or the experimental procedure (experiment 3).

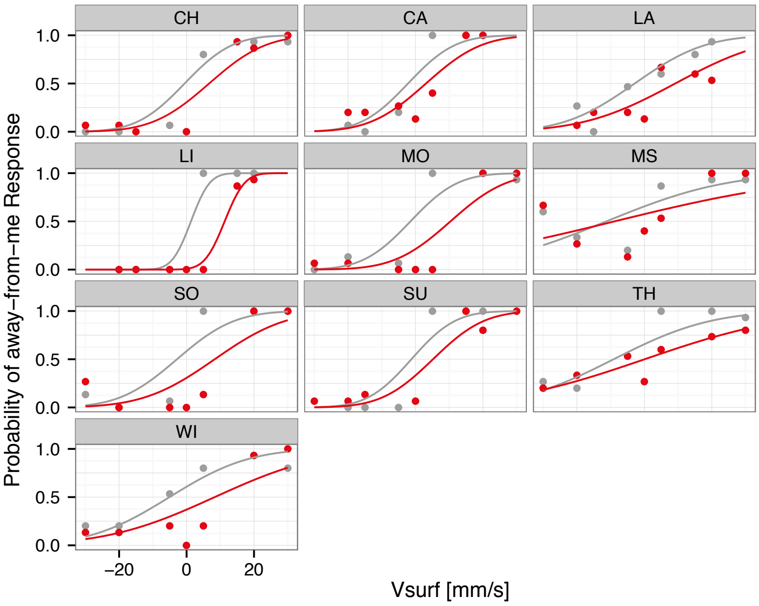

Supplementary Figure S2: Tactile Filehne Illusion (experiment 1). Raw data GLMM fit in the main and control condition. The main and control condition are plotted in red an in gray, respectively. The main task included also world-stationary stimuli, whereas v_{surf} was always different from zero in the control task ($v_{\text{surf}} = 0$ would have produced no tactile stimulus in the control condition). We replicated the population-level analysis excluding in the main task those trials were $v_{\text{surf}} = 0$, to verify that the bias task was not restricted to these ambiguous stimuli. The analysis confirmed the main result, namely that the 95% confidence intervals of the PSEs did not overlap between the main task than in control task, and were significantly larger than zero in the main task.

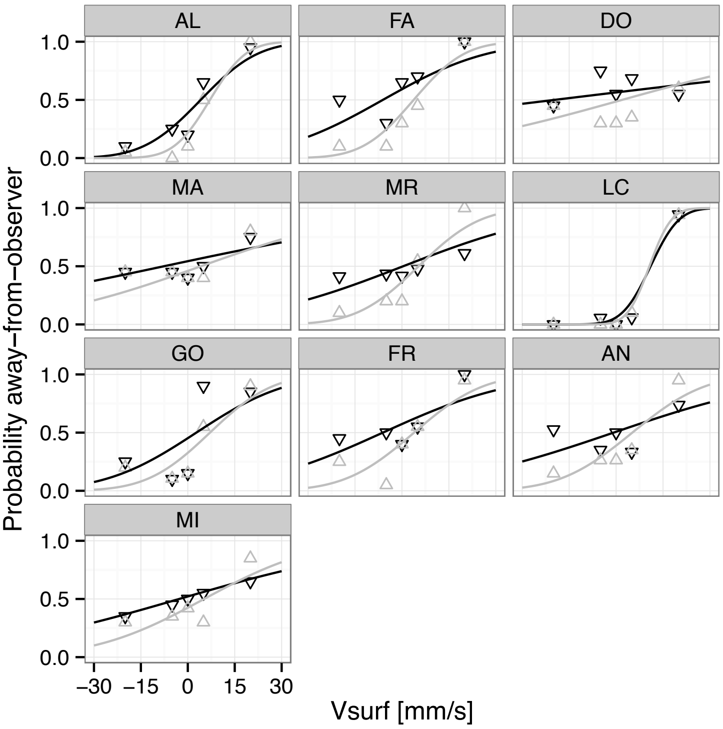

Supplementary Figure S3: Tactile Filehne Illusion (experiment 2). Raw data and GLMM. The low and the high oscillation amplitude condition are represented in dark gray and in light gray, respectively. The low amplitude condition is characterized by a noisier response (curves are shallower) and a smaller bias (curves are shifted on the x-axis) than in the high amplitude condition.

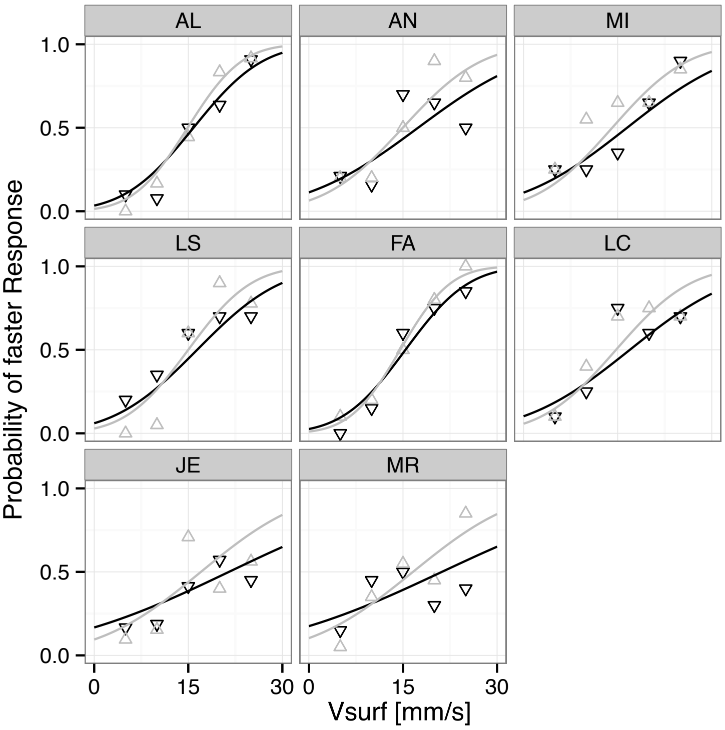

Supplementary Figure S4: Velocity discrimination task (experiment 3). Raw data and GLMM fit. In each trial, observers maintained the hand world-stationary and reported which of two subsequent stimuli was moving faster. The amplitude of pin oscillation changed between trials and was either 0.1 mm or 0.04 mm (high and low amplitude condition, respectively). The low and the high oscillation amplitude condition are represented in dark gray and in light gray in the figure, respectively. The two conditions were pseudo-randomly intermixed. Each experimental session consisted of 200 trials. The order of presentation of the standard and the comparison stimulus changed pseudo-randomly between trials. The difference in noise between condition was statistically significant ($p < 0.01$)

The Bayesian Model: From the Latent Variables to the Observed Responses

In the foregoing, the prior, the likelihood and the posterior distributions are named as in Fig. 3 of the article. We assume that the observed binary response Y_j originates from a continuous latent variable $\hat{v}_{\text{surf}}|v_{\text{surf}} = x$, which corresponds to the perceived velocity of the surface for a given value of its physical velocity $v_{\text{surf}} = x$. Please see [1] for a exhaustive discussion of the latent variable approach. The observed and the latent variables are related by

$$Y_j = \begin{cases} 1 & \text{if } \hat{v}_{\text{surf}} > 0 \\ 0 & \text{if } \hat{v}_{\text{surf}} \leq 0 \end{cases}$$

The latent variable is the algebraic sum of the proprioceptive-based and the tactile-based posterior estimates, corresponding to $\hat{v}_0$ and $\widehat{\Delta v}$ in our experimental design,

$$\hat{v}_{\text{surf}} = \hat{v}_0 + \widehat{\Delta v} \tag{S2}$$

Both $\hat{v}_0$ and $\widehat{\Delta v}$ are normally distributed random variables. We can write each of them as the sum of the mean of the distribution and zero-mean Gaussian noise,

$$\hat{v}_0 = \mu_{\hat{\text{v}}_0} + \epsilon_{\hat{\text{v}}_0} \tag{S3}$$

$$\widehat{\Delta v} = \mu_{\widehat{\Delta \text{v}}} + \epsilon_{\widehat{\Delta \text{v}}} \tag{S4}$$

where $\mu_{\hat{\text{v}}_0}$ and $\mu_{\widehat{\Delta \text{v}}}$ stand for the mean of the two posterior distributions. Then, the probability that $Y_j = 1$ is equal to:

$$\begin{aligned} P(Y_j = 1) &= P(\hat{v}_{\text{surf}} > 0) \\ &= P(\hat{v}_0 + \widehat{\Delta v} > 0) \\ &= P(\mu_{\hat{\text{v}}_0} + \epsilon_{\hat{\text{v}}_0} + \mu_{\widehat{\Delta \text{v}}} + \epsilon_{\widehat{\Delta \text{v}}} > 0) \\ &= P[(\epsilon_{\hat{\text{v}}_0} + \epsilon_{\widehat{\Delta \text{v}}}) > -(\mu_{\hat{\text{v}}_0} + \mu_{\widehat{\Delta \text{v}}})] \end{aligned} \tag{S5}$$

Assuming that the two noise terms $\epsilon_{\hat{\text{v}}_0}$ and $\epsilon_{\widehat{\Delta \text{v}}}$ are uncorrelated, we can standardize the two sides of the inequality,

$$\begin{aligned} P(Y_j = 1) &= P\left(\frac{\epsilon_{\hat{\text{v}}_0} + \epsilon_{\widehat{\Delta \text{v}}}}{\sqrt{\sigma^2_{\hat{\text{v}}_0} + \sigma^2_{\widehat{\Delta \text{v}}}}} > -\frac{\mu_{\hat{\text{v}}_0} + \mu_{\widehat{\Delta \text{v}}}}{\sqrt{\sigma^2_{\hat{\text{v}}_0} + \sigma^2_{\widehat{\Delta \text{v}}}}}\right) \\ &= \Phi\left(\frac{\mu_{\hat{\text{v}}_0} + \mu_{\widehat{\Delta \text{v}}}}{\sqrt{\sigma^2_{\hat{\text{v}}_0} + \sigma^2_{\widehat{\Delta \text{v}}}}}\right) \end{aligned} \tag{S6}$$

The four parameters $\mu_{\hat{\text{v}}_0}$, $\mu_{\widehat{\Delta \text{v}}}$, $\sigma^2_{\hat{\text{v}}_0}$, and $\sigma^2_{\widehat{\Delta \text{v}}}$ define the two posterior distributions of the Bayesian process considered in the main text. Since the mean of the prior S is equal

to 0, the means of the posteriors are linear functions of the physical velocities v_{prop} and v_{tact}, respectively

$$\mu_{\hat{\text{v}}_0} = \left(\frac{1/\sigma^2_{v_{\text{prop}}}}{1/\sigma^2_{v_{\text{prop}}} + 1/\sigma^2_S},\right)\mu_{v_{\text{prop}}} = \alpha_{v_{\text{prop}}}\mu_{v_{\text{prop}}} = \alpha_{v_{\text{prop}}}v_{\text{prop}}$$

$$\mu_{\widehat{\Delta\text{v}}} = \left(\frac{1/\sigma^2_{v_{\text{tact}}}}{1/\sigma^2_{v_{\text{tact}}} + 1/\sigma^2_S}\right)\mu_{v_{\text{tact}}} = \alpha_{v_{\text{tact}}}\mu_{v_{\text{tact}}} = \alpha_{v_{\text{tact}}}v_{\text{tact}}$$

These expressions result from the assumption that each of the two unimodal sensory estimate is unbiased, thus the mean of the likelihood distribution ($\mu_{v_{\text{prop}}}$ or $\mu_{v_{\text{tact}}}$) is equal to the physical velocity (v_{prop} or v_{tact}, respectively). The variances of the posteriors are

$$\sigma^2_{\hat{\text{v}}_0} = \frac{\sigma^2_{v_{\text{prop}}}\sigma^2_S}{\sigma^2_{v_{\text{prop}}} + \sigma^2_S}, \quad \sigma^2_{\widehat{\Delta\text{v}}} = \frac{\sigma^2_{v_{\text{tact}}}\sigma^2_S}{\sigma^2_{v_{\text{tact}}} + \sigma^2_S} \tag{S7}$$

Equation (S6) can be re-written in terms of the variance of static prior and of the two likelihood distributions,

$$P(Y_j = 1) = \Phi\left(\frac{\alpha_{v_{\text{prop}}}v_{\text{prop}} + \alpha_{v_{\text{tact}}}v_{\text{tact}}}{\sqrt{\left(\frac{\sigma^2_{v_{\text{prop}}}\sigma^2_S}{\sigma^2_{v_{\text{prop}}}+\sigma^2_S}\right) + \left(\frac{\sigma^2_{v_{\text{tact}}}\sigma^2_S}{\sigma^2_{v_{\text{tact}}}+\sigma^2_S}\right)}}\right) \tag{S8}$$

This expression has three free parameters, the three variances since the weighting factors are themselves function of the variances. For a constant value of $v_{\text{prop}} = v_0$, as in the main and the control task, the equation (S7) can be written as a function of v_{surf},

$$\begin{aligned}
P(Y_j = 1) &= \Phi\left(\frac{\alpha_{v_{\text{prop}}}v_0 + \alpha_{v_{\text{tact}}}(v_{\text{surf}} - v_0)}{\sqrt{\frac{\sigma^2_{v_{\text{prop}}}\sigma^2_S}{\sigma^2_{v_{\text{prop}}}+\sigma^2_S} + \frac{\sigma^2_{v_{\text{tact}}}\sigma^2_S}{\sigma^2_{v_{\text{tact}}}+\sigma^2_S}}}\right) \\
&= \Phi\left(\frac{\alpha_{v_{\text{prop}}}v_0 + \alpha_{v_{\text{tact}}}(v_{surf} - v_0)}{\sqrt{\sigma^2_{v_{\text{surf}}}}}\right) \\
&= \Phi\left(\frac{v_0(\alpha_{v_{\text{prop}}} - \alpha_{v_{\text{tact}}})}{\sqrt{\sigma^2_{v_{\text{surf}}}}} + \frac{\alpha_{v_{\text{tact}}}v_{\text{surf}}}{\sqrt{\sigma^2_{v_{\text{surf}}}}}\right) \\
&= \Phi(\beta_0 + v_{\text{surf}}\beta_1).
\end{aligned}$$

The intercept, the slope, and the PSE of the psychometric functions are

$$\beta_0 = \frac{(\alpha_{v_{\text{prop}}} - \alpha_{v_{\text{tact}}})v_0}{\sqrt{\sigma^2_{v_{\text{surf}}}}}$$

$$\beta_1 = \frac{\alpha_{v_{\text{tact}}}}{\sqrt{\sigma^2_{v_{\text{surf}}}}}$$

$$\text{PSE} = -\frac{\beta_0}{\beta_1} = -\frac{(\alpha_{v_{\text{prop}}} - \alpha_{v_{\text{tact}}})v_0}{\alpha_{v_{\text{tact}}}}$$

The predicted PSE is positive for $v_0 > 0$ and $\alpha_{v_{\text{tact}}} > \alpha_{v_{\text{prop}}}$, and equal to zero for $v_0 = 0$, i.e. the finger is not moving, as in the control task, or $\alpha_{v_{\text{tact}}} = \alpha_{v_{\text{prop}}}$, i.e., the detection of the velocities are equally reliable. Also, it is worth noting that the variance of the posterior of the combined estimate $\sigma^2_{v_{\text{surf}}}$ cannot be defined from the slope (or the JND) of the psychometric function, due to the weight term depending on σ^2_S. The same identifiability problem would arise in a single-cue discrimination task.

Note that, in the model proposed in [9, 4], the estimate is the maximum a posteriori (MAP), corresponding to the mean or the mode of the posterior. This would produce a deterministic response. In order to account for the variability of the response between trials, Stocker and Simoncelli [9] proposed to combine the mean of the likelihood with a separate source of internal noise. Instead, in (S3) and (S4) we assumed the estimate to be a random sample from the posterior distributions. This way, the model did not require the further level of randomness to account for the variability of the response.

Noise in tactile and proprioception velocity perception: Data from the literature

The Bayesian model assumes that the tactile velocity measurement $\hat{v}_{\text{tact}}$ is less noisy than the proprioceptive velocity measurement $\hat{v}_{\text{prop}}$ and that each of the two measurements is unbiased.

Bensmaïa et al.[2] measured the tactile velocity discrimination threshold, using a sinusoidal grating with a period of 8 mm and a reference velocity of 40 mm/s. The estimated velocity was unbiased (PSE = 39.3 ± 6 mm/s, mean±SE). The JND was 6.5±0.88 mm/s, yielding to a Weber fraction of 0.16. Kerr and Worringham measured the proprioceptive discrimination threshold for a movement restricted to the elbow joint [5]. They reported a discrimination threshold ranging from 4.5 to 10 °/s for different reference velocities ranging from 15 to 75 °/s. Thus, the average Weber fraction was 0.24. Lönn et al.[6] and Djupsjöbacka and Domkin [3] measured the velocity discrimination threshold for a movement restricted to the glenohumeral joint. The estimated Weber fraction varies between the two studies and between different ranges of velocities tested with an average of 0.13. Moscatelli et al.[7] compared the precision of the response between a tactile and a proprioceptive velocity discrimination tasks. In the proprioceptive-based task, the required movement involved the elbow and wrist joints. Weber fractions were 0.17 for touch and 0.18 for proprioception.

In summary, the ratio between the tactile based and the proprioceptive based noise ranges from 1.2 (proprioceptive discrimination based on the shoulder joint movement) to 0.64 (elbow joint movement) and 0.67 (two-joint movement). Overall, this ratio is different from the 1:3 ratio predicted by the Bayesian model by a factor of 2 or more.

References

[1] D. J. Bartholomew, M. Knott, and I. Moustaki. *Latent variable models and factor analysis: A unified approach*, volume 899. Wiley, 2011.

[2] S. J. Bensmaia, J. H. Killebrew, and J. C. Craig. Influence of visual motion on tactile motion perception. *Journal of Neurophysiology*, 96(3):1625–1637, 2006.

[3] M. Djupsjöbacka and D. Domkin. Correlation analysis of proprioceptive acuity in ipsilateral position-matching and velocity-discrimination. *Somatosensory and Motor Research*, 22(1-2):85–93, 2005.

[4] T. C. A. Freeman, R. A. Champion, and P. A. Warren. A bayesian model of perceived head-centered velocity during smooth pursuit eye movement. *Current Biology*, 20(8):757–762, 2010.

[5] G. K Kerr and C. J. Worringham. Velocity perception and proprioception. In *Sensorimotor Control of Movement and Posture*, pages 79–86. Springer, 2002.

[6] M. Lönn, H. Djupsjöbacka, and J. Johansson. Replication and discrimination of limb movement velocity. *Somatosensory & Motor Research*, 18(1):76–82, 2001.

[7] A. Moscatelli, G. Scheller, G. J. Kowalski, and M. O. Ernst. The haptic analog of the visual aubert-fleischl phenomenon. In *Eurohaptics Conference*. EHS, Springer, 2014.

[8] J. C. Pinheiro and D. M. Bates. *Linear mixed-effects models: basic concepts and examples*. Springer, 2000.

[9] A. A. Stocker and E. P. Simoncelli. Noise characteristics and prior expectations in human visual speed perception. *Nature neuroscience*, 9(4):578–585, 2006.
